# Effects of COVID-19 pandemic on provision and use of maternal health services in Allada, southern Benin: a local health system perspective

**DOI:** 10.3389/fpubh.2023.1241983

**Published:** 2023-11-16

**Authors:** Éric Akpi, Armelle Vigan, Christelle Boyi Hounsou, Marlène Gandaho, Gisèle Houngbo, Charlotte Gryseels, Jean-Paul Dossou, Thérèse Delvaux

**Affiliations:** ^1^Centre de Recherche en Reproduction Humaine et en Démographie (CERRHUD), Cotonou, Benin; ^2^Institute of Tropical Medicine (ITM), Antwerp, Belgium

**Keywords:** maternal health care, family planning, childbirth, COVID-19, local health system, Benin

## Abstract

**Objective:**

To assess the effects of the COVID-19 pandemic on the provision and use of maternal health services in southern Benin from a local health system perspective.

**Methods:**

We conducted a qualitative study from April to December 2021 in a health district in southern Benin. We interviewed health workers involved in antenatal, delivery, postnatal and family planning care provision, alternative and spiritual care providers, administrative staff of the district hospital, community health workers, adolescents and women who had given birth in the past six weeks in public health centers were interviewed. The World Health Organization health systems building blocks framework was used to guide the thematic analysis from a local health system perspective.

**Results:**

The COVID-19 pandemic changed the lines of command and the institutional arrangements in the local health systems leadership; it put the interpersonal relationships in the health care provision team under stress and reduced the overall revenues of the district hospital. The motivation of allopathic health workers was undermined. Communities underutilized maternal health services in the COVID-19 period. Plausible causes included negative patient perceptions of COVID-19 measures taken at the public health facility level as well as well as fear of being forcibly vaccinated against COVID-19 in the health facilities.

**Conclusion:**

In times of health crises, appropriate local health system governance that integrates providers’ concerns into effective guidelines is critical to reach and maintain a sufficient level of work motivation to ensure quality maternal health services.

## 1. Introduction

The COVID-19 pandemic has further constrained already overburdened and underfunded health systems to respond to critical health care concerns (1). Research had already shown that emerging epidemics in sub-Saharan Africa can lead to reduced access to maternal health care services, particularly in health systems that lack responsiveness or resilience (2). In Zimbabwe, due to the COVID-19 pandemic, the number of cesarean sections performed between January and April 2020 decreased by 42% compared to the same period in 2019; and the number of live births in health facilities and the number of contraceptive pill users decreased by 21% and 90%, respectively. In Burundi, early pandemic statistics showed that births assisted by skilled health personnel decreased from 30,826 in April 2019 to 4,749 in 2020 (3). In Kenya, Uganda, and Tanzania, midwives reported low numbers of people attending maternal health clinics and more women arriving late in labor at hospitals without having received adequate antenatal care (3). The study by Semaan *et al* found a significant reduction of the use of antenatal care services as clinics reduced operating hours, number of visitors and frequency of visits allowed (4). In the same line, another study of health care utilization and maternal and infant mortality in 18 low- and middle-income countries based on interrupted time series analysis with mathematical modeling of service data found that the largest disruptions, associated with 27.5% of excess deaths, occurred in the second quarter of 2020, regardless of whether countries reported the highest rate of COVID-19 mortality in the same months (5). Pandemic-induced restrictions were among the reasons reported to explain these findings (5). In addition, maternal care users chose to forgo their visits due to fears about the virus and rumors about the origin of the pandemic, which led to mistrust of health care workers and confusion about what services were still available (6, 7). Health services have also been disrupted by the COVID-19 pandemic in Benin. A recent study showed that at the local or health district level hospital managers had to operate in a complex and dynamic environment, but with limited decision-making power due to the high level of uncertainty in terms of resources and environment (8). However, knowledge gaps remain about the systemic effects of the COVID-19 pandemic on the various dimensions of local health systems, including the (re) organization of maternal health care provision at the level of the health districts. The objective of this study was to assess such effects of the COVID-19 pandemic on the provision and use of maternal health services in southern Benin from a local health system perspective (9)

## 2. Methods

### 2.1. Study site and population

This qualitative study was conducted in a health district located in the Atlantic department (southern Benin), the most densely populated department in the country with 1,398,229 inhabitants in 2013 (9). The health district is made up of three communes, from which the main one is considered as the historical and cultural epicenter of the *Fon* ethnic group, representing approximately 40% of the population of Benin (10). The indigenous *Vodun* religion provides the main spiritual framework, alongside Christianity and Islam (11).

#### 2.1.1. Pre-pandemic local health system

In 2018, the health district benefited from a new referral hospital built through an international bilateral cooperation with Japan. Its maternity ward had a capacity of 18 beds and offered a full range of maternal health services including antenatal care (ANC), normal delivery, caesarean section, postnatal care (PNC), family planning (FP), ultrasound services with a team of gynecologists, midwives, and nurses. Located in Allada city and 8,8 km away from the health district hospital, the health center of the main commune is an older facility, much less spacious than the district hospital. It comprised a 15-bed maternity ward with a delivery room, two hospitalization rooms, and a duty room. Two midwives and a state nurse constitute each duty shift. ANC, normal delivery, PNC, and FP consultations but no ultrasound services were available at this communal health center. Close to the health center, there is another vertical program health facility dedicated to the screening and holistic treatment of Buruli ulcer. That facility had a laboratory, an X-ray service, and a well-equipped technical platform for reconstructive surgery.

#### 2.1.2. Pandemic-induced changes to the health system

During the pandemic, the district hospital was transformed into one of the main quarantine and treatment centers for COVID-19 in southern Benin (Table 1). The communal health center was then adapted to temporarily serve as the district referral hospital. This temporary “district hospital” had a capacity of 31 beds including the maternity ward and was equipped with an ultrasound machine transferred from the district hospital. The maternity staff of the district hospital were also transferred to the maternity ward of the communal health center. The district hospital’s surgical activities (including obstetrics and gynecology) and laboratory services were transferred to the nearby Buruli ulcer center. Antenatal care (ANC), postnatal (PNC) care and family planning (FP) services, which were previously offered by the communal health center, were now only available in the peripheral health centers of the health district (See Supplementary Table S1).

**Table 1:** Changes induced by the COVID-19 pandemic to the local system infrastructures in Allada health district, Southern Benin, 2018 to 2022.

	Before COVID-19 (2018)	During COVID-19 (2020 and 2021)	After COVID -19 follow-up (2022)
Main health facilities in Allada	1 District Hospital	Became the COVID-19 treatment center*	1 District hospital
	1 Communal Health Center	Became the temporary District Hospital*	1 Communal Health Center
	12 public peripheral health centers, 2 private health centers, 1 faith-based health center	11 public peripheral health centers, 2 private health centers, 1 faith-based health center	12 public peripheral health centers, 2 private health centers, 1 faith-based health center

*In bold, major changes that occurred during Covid-19 pandemic.

#### 2.1.3. Clinical guidelines during the pandemic

Polymerase Chain Reaction (PCR) testing was performed at the temporary district hospital or a peripheral public health center only if there were clinical signs suggesting a COVID-19 infection. When positive but in the absence of severe signs (mild symptomatic COVID-19), providers would start treatment and advise home confinement, reminding the patient of the severe signs (COVID-19 – and pregnancy-related) that would bring her back to the hospital. In case of serious symptoms (severe COVID-19), the patient was directly referred to the COVID-19 treatment and care center. The providers also described two specific pathways for women in labor: women who came to the temporary district hospital or a peripheral health center and tested negative gave birth in the facilities without being referred to the COVID-19 treatment and care center. However, some women had to be transferred to the hospital in the neighboring health district because there was no availability in the operating theatre of the temporary district hospital. The second pathway was for those whose PCR test was positive: they were transferred by ambulance to the COVID-19 center and taken care of by the gynecologists of the COVID-19 control team.

### 2.2. Data collection and management

Data were collected between September and December 2021. Researchers with a background in social science and public health used qualitative research techniques, including in-depth interviews, informal conversations, group discussions, and observations. We used the World Health Organization (WHO) health system building blocks framework including the six essential health system building blocks with their respective functions, namely leadership and governance, service delivery, health system financing, health workforce, medical products, vaccines, and technologies, health information systems and community (12) to initially structure the interview guides. Nevertheless, open-ended questions allowed for the spontaneous emergence of new information and subsequent adaptation of the question guide. All interviews were conducted in either French or *Fon*. The research team transcribed the verbatim recordings of the interviews and translated them to French. Observations (including informal conversations) were conducted in public and private health facilities and the community using an observation grid. A summary of data was produced for each observation and used for analysis along with the transcribed interview data.

### 2.3. Sampling

Theoretical sampling, which consists of simultaneously collecting and analyzing data using existing theories to guide the next steps of the research and the selection of new participants, was used. The participants included in the study were health workers involved in maternal health services (maternity wards) in the health district (*n* = 23), alternative and spiritual care providers (*n* = 8), hospital administrative staff (*n* = 6), community health workers (*n* = 4), and women who had delivered in the last six weeks in public health facilities (*n* = 26). These participants were identified in 10 health facilities, including peripheral public health facilities (*n* = 7), a public district hospital (*n* = 1), a private health facility (*n* = 1), and a faith-based health facility (*n* = 1). Alternative and spiritual care providers were identified within the community using the snowball sampling technique.

### 2.4. Analysis

#### 2.4.1. Qualitative data

**Figure 1:**
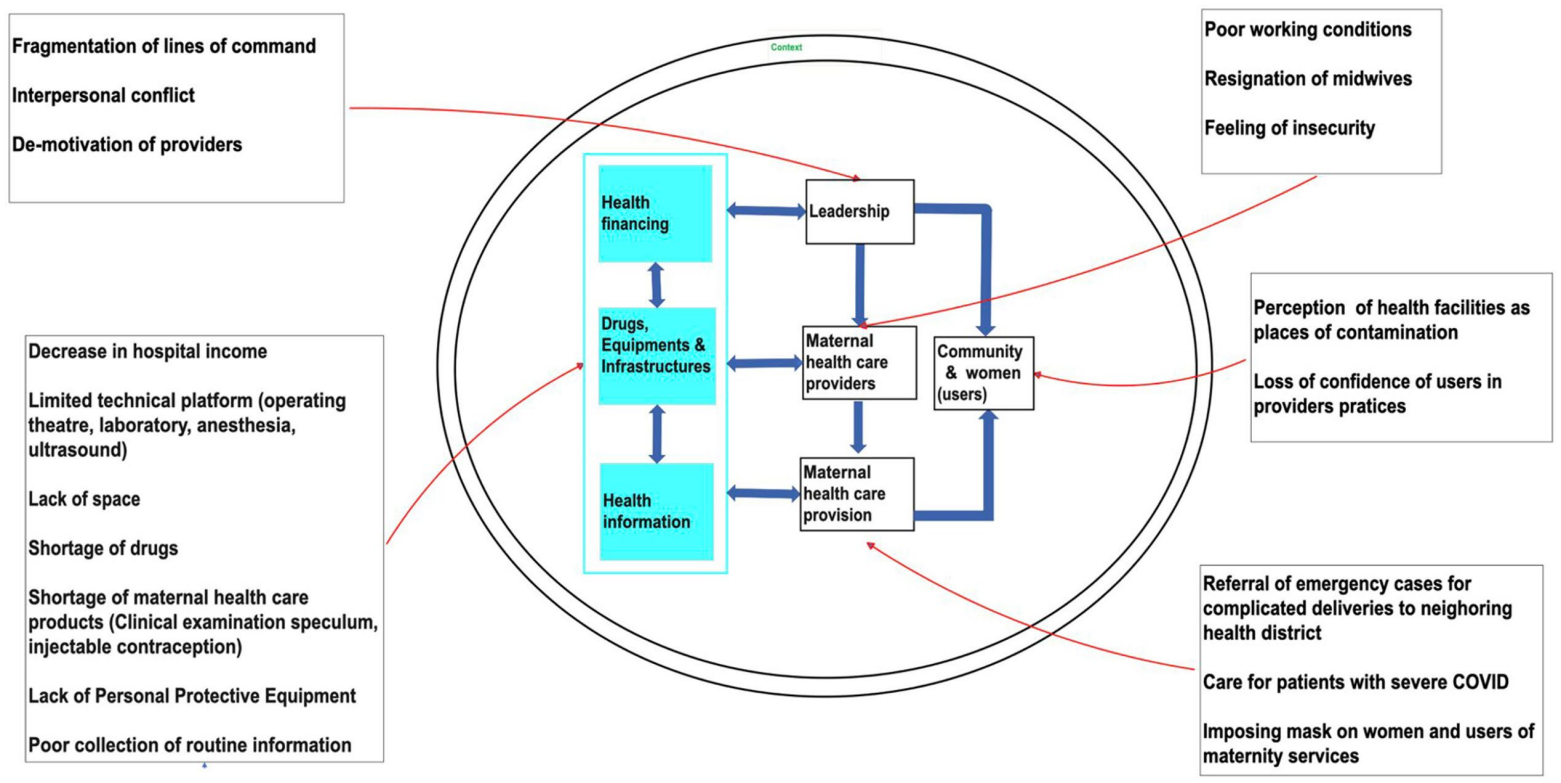
Barriers to delivering and accessing quality care using the WHO health system building blocks.

Thematic analysis was a flexible and iterative process. Preliminary analysis was conducted while new themes emerged until saturation was reached. All transcripts were analyzed to identify key themes, which were validated by the research team and used to develop codes and a subsequent analytical framework (Figure 1). Dedoose version 9.0.54 qualitative data analysis software was used for analysis.

#### 2.4.2. Quantitative data

Data analysis also relied on birth statistics from the local health system. These data were first extracted manually from the records of each health facility included in the study and then compared with those available on the District Health Information System version 2 (DHIS2) to verify their accuracy. They were tabulated in an excel file and then graphically presented. They were used to compare the number of deliveries at the community health center and the district hospital before, during, and after the response activities in the local health system.

## 3. Results

The first section of the results will describe the pandemic-induced barriers providers experienced in delivering quality care, while the second section describes the barriers to accessing quality care from the point of view of community members. Figure 1 illustrates the barriers from both sides using the WHO building blocks. The last section shortly describes the situation after the pandemic.

### 3.1. Reported barriers to delivering quality care

#### 3.1.1. Shortages in equipment and drugs

An increased awareness for the need of personal protective equipment (PPE) to protect themselves – and the lack thereof – was the first difficulty reported by providers during COVID-19 pandemic. The stress caused by the lack of PPE and other materials was reported to be negatively impacting the quality of care they delivered. There was also a significant loss of equipment and consumables in the transfer of the well-equipped district hospital to the communal health center (gynecologic examination speculum, IUD removal material). Managers mentioned that loss of equipment is due to the lack of preparation and coordination of the transfer of equipment from the district hospital site to the community health center. In addition, the new working conditions in the temporary district hospital contributed to the damage and subsequent malfunction of the ultrasound machine. One provider described:

“The ultrasound room [ultrasound] has no air-conditioning so the windows are open to allow for ventilation. The gynecologist was in the theatre when the rain started. The rain got the ultrasound machine wet; she had forgotten that she had left the windows open, and it was when she finished the cesarean, she went back, she saw that the machine could no longer be turned on.” (In-depth interview, provider)

Providers working in the surgical theatre mentioned that the provision of oxygen at the temporary district hospital was disrupted, especially during the peak of the third COVID-19 wave, due to the high demand at the COVID-19 treatment and management center. The temporary district hospital department also lacked Intrauterine Device (IUD) removal equipment as existing (gynecologic examination) specula were rusty and outdated. Although providers reported shortages were already a problem, the COVID-19 pandemic exacerbated this shortage of health care equipment, which the government had sought to address by providing the health district with a brand-new hospital in 2018. Most of the periphery health centers also regularly recorded long shortages of FP products and lacked the necessary equipment to remove IUD methods. For example, 4 of the 14 health centers visited had a Sayana Press (injectable contraception) stock out from July to November 2021.

#### 3.1.2. Priority setting due to lack of space and time

Due to the reorganization of the health services during the pandemic, midwives reported prioritizing ANC and deliveries over PNC and FP consultations. They felt they had less time to spend per patient, which severely affected their perceived quality of PNC and FP services. The lack of space in the temporary district hospital (former communal health center) also reduced the perceived quality of care they could provide. A provider recalled the organizational difficulties encountered in the management of obstetrical emergencies at the temporary district hospital due to lack of an internal walkway connecting the two spaces:

“When it comes to operating on a pregnant woman, the patient must be put in the ambulance with the midwife to “go around” to operate. Yesterday, for example, they wanted to perform a caesarean section and the ambulance was on its way to the University Hospital (Cotonou); the parents had to look for a motorcycle to put the patient on it to bring her to the other side [operating theater building] (…). I’ve had to put a patient dripping with blood in my car to transport her to the other side because she cannot stay on a motorcycle. It was the middle of the night. There was no ambulance, that’s it…” (In-depth interview, gynecologist)

For the supervisors, lack of space led to feelings of discomfort. The offices were transformed into an on-call room or a consultation room at the maternity ward level. One provider explains:

“[…] so let us say that there is no office for the head of department, there is no office for the maternity supervisor as such can the office in which she is placed, this is where she still receives the CPoN and the PF […] (*In-depth interview, provider*)

Providers also reported these changed working conditions led to less consistency in respecting immediate postnatal consultations, as illustrated by another provider:

“There is no harmonization as such because of all this there, (…) [normally] you have to send people to the postpartum ward, and they stay there, we monitor them, now because of the space, we are forced to bend the normal procedure” (In-depth interview, provider).

The obstetric care protocol normally promotes postnatal monitoring from the first two hours to 7 days after delivery. Not being able to respect this monitoring phase was reported to also be due to the COVID-19 testing and isolation procedures in public health facilities. The diagnosis of COVID-19 was primarily made for women when they visited the health facility for ANC or when they were admitted for childbirth. Health providers report that in the beginning of the implementation of response measures, anyone detected with flu symptoms and fever in peripheral health centers or in the temporary district hospital was systematically admitted to the COVID-19 treatment center, severely complicating their ability – in terms of time and space – to adhere to medical protocols.

In addition, the length of stay in the public health facilities and in the hospital became shorter (less than 72 h for normal deliveries at hospital, for example).

“[…] It is that there are patients that we release a lot rather for example women after a C-section theoretically should do 7 days in the hospital with their children. Because of the space, and the number of women who come to give birth, we released them at most on the 5th day, but if we really need space already at 3 days while women after a vaginal birth should do 2 or 3 days minimum, but we release them in 24 h […].” (In-depth interview, provider)

#### 3.1.3. Staffing shortages and workload

According to providers, staffing shortage and increased workload in maternity wards during the pandemic were related to two main causes. First, the two-week quarantine given to providers in case of a positive COVID-19 test reduced staffing. Providers were called to be tested in case of flu signs or contact with a pregnant woman or patients who had a positive COVID-19 test, especially since they were not sufficiently equipped with Personal Protective Equipment (PPE) in every care provision interaction. This caused fear of not being able to properly care for patients and of getting infected themselves, leading to stress. Secondly, the providers were actively involved in the COVID-19 response activities as members of the rapid response team and the community brigade for sensitization or involved in COVID-19 vaccination in addition to their classic workload. The gynecologists working in the temporary district hospital also followed up and delivered babies in the COVID-19 treatment and care center. In addition, their on-call schedules were changed. This has led to an increase in workload:

“The three gynaecologists were on duty three times each a week [in the district hospital]. The gynaecologist on a service contract only came to work on weekends […]. Now, the same gynaecologist must do a permanence followed by on-call to cover. For example, when someone is on call, he alone manages the technical block, the delivery room, the operating room” (In-depth interview, manager).

Consequently, providers reported that approximately one-third of hospital staff had resigned during the COVID-19 crisis.

#### 3.1.4. Reductions in income

The reduction in hospitalization capacity and in several technical procedures due to the transfer of activities to the communal health center, as well as the loss of patients in general due to COVID-19 pandemic, resulted in a significant drop in the temporary district hospital’s income. This has altered both the hospital’s ability to motivate providers, especially at the maternity ward, and the quality of care due to difficulties in acquiring equipment such as PPE and specula. Managers also reported a loss of revenues due to an unequitable share of the revenue coming from the services offered using the platform of the Buruli ulcer treatment center. On that note, a manager reported:

“We agreed with a 60% split for the hospital and 40% for the Buruli ulcer center! […] what they told us to convince us a little is that the equipment belongs to them. They will take care of the equipment maintenance and depreciation, (…) as if their equipment breaks down, it will be at their expense.” (In-depth interview, manager)

This agreement was applied to all revenues obtained from common services, the surgical theatre and the laboratory.

#### 3.1.5. Fragmented leadership, conflict, and dialogue

The management team of the former communal health center that was transformed into district hospital was relocated to the nearby Buruli ulcer program, causing additional challenges in coordinating and maintaining maternity services. Challenges included reported loss of autonomy due to fragmented lines of command at the operating theatre, which now had to be shared by gynecologists of the temporary district hospital and surgeons of the vertical program health facility, leading to conflicts between doctors. Other challenges reported included inadequate management of health providers’ concerns about being infected during births and lack of trained staff due to sick leave and quarantine measures. Despite planned training for COVID-19 case management, the lack of a clearly defined protocol and sufficient PPE to minimize the risk of contamination during births brought out feelings of fear, stress, and demotivation. Faced with these challenges, managers created spaces for dialogue and consultation with the care teams to encourage collaboration. From the providers’ point of view, this initiative contributed to strengthening cohesion and leadership within the care teams, which helped to overcome staff demotivation resulting from poor working conditions.

### 3.2. Reported barriers to accessing quality care

#### 3.2.1. Community perceptions of testing and isolation measures

The pandemic and the response measures implemented in the local health system first gave rise to the perception that health facilities are places where people could be infected. When COVID-19 vaccines became available, these were perceived to be incorporated into modern injectable FP methods and aimed at destroying fertility. Informants from surrounding communities report that they saw providers as the operational arm of a conspiracy between the central state and “white people” who also sent the AIDS vaccines to Africa to decimate the African population.

“When we go there to give birth, we are asked to at least put on the mask […]. But we are not convinced. This is how they think they are infecting us like they did with condoms for AIDS [did you learn that?] and our families will not even have access to our bodies.” (In-depth interview, woman giving birth)

This perception, widely shared by community members, fueled vaccine hesitancy, led to a loss of confidence in the practices of health care providers in the local health care system, and increased communities’ reliance on alternative and spiritual care. Informants further report that this caused them to prioritize private health facilities for the use of maternal health care and services, which did not impose compliance with testing and isolation measures on users.

“As for attendance at the [public] health center, it has completely dropped. People prefer to go to private facilities that do not require barrier measures. Rumors circulate in the population according to which the agents [of the public health facilities] would require the health pass for all users. This caused them to flee to often unauthorized private clinics” (In-depth interview, provider)

Both community and provider informants perceive such perceptions to be the main reason behind the low uptake of maternal care in public health facilities during the pandemic.

“As soon as we go to the public health center, it is COVID-19 that the health workers diagnose us with. If you have a simple fever or a cough, the ambulance comes to take you to the COVID-19 center in Allada […] Even for childbirth, we do it at home” (In-depth interview, woman giving birth).

Health care providers reported seeing fewer women in public health facilities for antenatal consultations, deliveries, postnatal consultations, or FP, which they also considered a result of the fear women had of being found positive or even being injected with the COVID-19 vaccine while admitted in the public health facility. One provider recalled:

“I met a woman whose daughter had just given birth and is still in the operating room of the hospital. She came up to me and said, I went here with a lot of courage because everyone was advising me not to come, saying that you give the vaccine to all the people [who come here],” (In-depth interview, provider)

#### 3.2.2. Drug shortages

The availability of drugs and products for delivery was also disrupted at the temporary district hospital. As a result, family members accompanying women in labor often received prescriptions at late hours for drugs which were no longer available in the hospital and they had to obtain them from private pharmacies in the city. Some informants reported obtaining the required drugs and other medical products from the informal drug market.

“[…], in this case, women go to the drug (informal) sellers and even though it’s forbidden, these sellers are accessible, particularly in remote areas, so they go and buy pills from those who sell in the informal sector […]” (In-depth interview, woman giving birth).

This alternative health seeking behaviour must be understood in a context where the informal drug market was heavily repressed with state actions for almost a decade prior to the pandemic. This practice includes imprisonment as well as payment of fines for sellers. However, the informal drug market remains available and accessible to overcome systemic dysfunctions in the availability of drugs during the pandemic.

Informants also reported an increase in indirect costs, such as unplanned transport to travel from one pharmacy to another, when delivering care to women in childbirth during the pandemic, which put a heavy burden on already struggling families.

#### 3.2.3. Financial challenges

Although providers were also struggling with reduced revenues, they were aware that their patients were having a hard time too. The temporary district hospital opted for a policy of reducing delivery costs to adapt the cost of health care to the financial ability to pay of the population using the health center. Community members reported this measure to be positive as it provided access to healthcare not only at a lower cost, but also of better quality than the maternal health care provided in the peripheral health centers.

### 3.3. After COVID-19 – recovery of the local health system

In March 2022 the operations of the COVID-19 treatment and care facility ended, and the district hospital returned to its premises. Towards the end of the pandemic (March to July 2022), a trend could be observed toward an increase in deliveries at both the district hospital and the community health facility (although the total was still lower than before COVID-19) (see Supplementary Figure S1). According to local health system managers, the transfer of the district hospital, with its superior technical platform, to the communal health center, also brought benefits for maternal first line care. The technical skills among midwives increased; the proximity of ultrasound and surgery (cesarean section) services improved care; the closer geographical proximity for many surrounding communities; and the policy of reducing the cost of services, improved the attendance rate at district hospital as soon as it was reinstated on its former site and at the communal health center as soon as its operations resumed.

## 4. Discussion

Using the WHO health system building blocks framework to guide the data collection, this study documented the effects of the COVID-19 pandemic on the provision and use of maternity services in a context where the local health system was modified to contain the epidemic in Benin.

### 4.1. Reorganization of health services delivery platform

The reorganization of maternal health services made it possible to set up two patient care circuits: a conventional circuit and a new circuit dedicated to women who test positive and have severe symptoms. Ensuring access to quality care for patients by guaranteeing their isolation while maintaining the usual circuit (4, 13–15) Maternal health services were maintained due to a shift in the lines of care downwards in the local health system, i.e., the relocation of most maternity services from the district hospital to the communal health center. This measure has led to profound changes in the care platform in the local health system, resulting in the creation of a “new” district hospital, with a substantially reduced technical platform. In Mali, buildings were also requisitioned to cope with the multiple effects of COVID-19 (16). In Ethiopia, some health facilities that provide TB care and treatment services were transformed into COVID-19 isolation and treatment centers (17) like in Benin.

### 4.2. Human resources challenges

We show that lack of qualified staff, lack of personal protective equipment, and lack of training and space, have had a direct impact on the quality of maternal care services providers felt they could deliver. Other studies have pointed to similar challenges in referral maternity hospitals in sub-Saharan Africa during the pandemic (4), which also extended to other types of care in hospitals in Kenya (18), Ethiopia (19, 20), Nigeria (21), Liberia (22, 23), South Africa (22), and even in Iran and the United Kingdom (24, 25). The lack of training on how to deal with epidemics was found to be a problem for all providers at all levels of the health system, including primary care physicians (26). This may be the result of the Beninese health system’s lack of pandemic preparedness strategies.

Our results further show that precarious working conditions due to such material challenges have led to provider demotivation. Consistent with this finding, a study in Mali showed that lack of PPEs and human resources were associated with a high risk of mental health disorders among providers at the beginning of the pandemic (27). The lack of materials is, however, inherent to the Benin’s healthcare systems, but was exacerbated by the COVID-19 pandemic (28). This led to a further decline in the quality of maternal services due to reduced consultation time and the prioritization of some care services (e.g., childbirth) over others (e.g., PNC and FP). The link between quality of care and provider demotivation has been found in studies on other types of primary care providers in Benin and Uganda (8, 29). An exacerbation of the already precarious working conditions could lead to the deterioration of their mental health and consequently, the quality of care they can provide (30). This, consequently, suggests that in situations of major health crises, psychological support for health care providers should be considered to support their mental health and maintain an acceptable quality of care.

### 4.3. Reductions in income

Our study shows that the income of the temporary district hospital was reduced due to the reduction in hospitalization capacity. This is a direct impact of COVID-19 control measures on hospital finances in many hospitals around the world (31, 32). The other reason in our context was the limited technical platform due to the transfer of activities from the district hospital to the communal health center. In line with this, Kouanfack et al., explore the effect of the pandemic on the financial performance of the Central Hospital of Yaoundé, Cameroon and show that the COVID-19 crisis has led to a decline in income from consultations and other types of health services (33). However, the hospital received donations and grants during the pandemic period which enabled the hospital to improve its technical facilities for better patient care.

### 4.4. The impact of leadership skills

While local system managers have embraced the national vision for combating the pandemic, the relocation of the district hospital and its cohabitation with the Buruli ulcer center have led to a confrontation between the health facilities. Old governance and leadership practices in the local health system were put to the test. The challenges raised by the pandemic in the governance and leadership of the local health system should provide a learning opportunity for the stakeholders involved, with the aim of strengthening the leadership skills of managers (34). A type of transformational leadership could have a significant positive impact on the motivation of workers in a context where the lack of protective equipment generates the fear of being infected. For example, during the severe acute respiratory syndrome (SARS) crisis of 2002–2003, Taiwanese charge nurses faced challenges similar to those described in our results (e.g., coping with shock and chaos, developing and adapting nursing care, supporting nurses and their clients, etc.). Through this kind of leadership, they developed an emotional support system not only for all providers, but also for service users (35). This system has been an important resource for managing interpersonal conflicts in their interactions with each other and with families.

### 4.5. The importance of maintaining public trust through community involvement

Regarding the maternal health service utilization, this study identified fear of vaccines and the virus as a factor reducing hospital attendance. Our informants expressed concerns that public health facilities might expose them to infection and even intentional injection with the virus, a sentiment observed in Nigeria and Pakistan as well (21, 36, 37). Numerous studies carried out at the beginning of COVID-19 vaccination campaigns in Sub-Saharan Africa also report fear of vaccines (38–40), which could be attributed to negative perceptions surrounding potential side effects of vaccines, distrust in the laboratories that created the vaccines, doubts about the distribution channels, and skepticism about vaccine effectiveness (39).

Our findings underscore the importance of actively listening to and engaging in a meaningful dialogue with communities to address their apprehensions regarding the implementation of public health measures. These concerns directly impact healthcare uptake. Previous epidemics have demonstrated that the influence of rumors and concerns on healthcare utilization during health crises can be mitigated through community engagement strategies and by taking community belief systems into careful consideration. For example, in the context of the Ebola crisis from 2013 to 2016, community involvement in two poor urban communities in the slums of western Sierra Leone made it possible to develop and deploy effective strategies such as control and monitoring strategies designed by community members (41). In addition, the strong involvement and ownership of community members in the government response improved their ability to generate resources to support local actions and to share appropriate information about the disease. These strategies have helped to contain the epidemic, improve access to health resources and services, and increase knowledge of effective Ebola prevention.

### 4.6. Limitations

This study was primarily qualitative in focus. We did not document the number of maternal deaths that occurred during the period of transfer of maternity services to the health facility site to compare them to data from the pre-COVID-19 period, nor did we document the number of pregnant women cared for at the COVID-19 health facility. However, these qualitative data illustrate the effect of the COVID-19 pandemic on maternal health services in a health district, which accomodated one of the country’s major COVID-19 treatment and care facilities.

## 5. Conclusion

The advent of the pandemic and the responses of the Beninese national health system have led to disruptions in the provision and use of maternal health services, where structural challenges were already limiting access to and quality of maternal health care. In times of health crises, appropriate local health system governance that integrates providers’ and communities’ concerns into effective guidelines are critical to reach and maintain a high level of motivation at work, a basic quality of maternal health services and assure continued hospital attendance of pregnant women and women in labor.

## Data availability statement

The original contributions presented in the study are included in the article/Supplementary material, further inquiries can be directed to the corresponding author.

## Ethics statement

The study involving humans was approved by the Institutional Review Board of the Institute of Tropical Medicine in Antwerp (REF. 1514/21 of 21/06/21), by the local ethics committee for biomedical research of the University of Parakou (REF. 0427 of 06/07/21) and an administrative authorization (N° 4601/MS/DC/SGM/DRFMT/SA of 13/10/2020) from the Ministry of Health. The study was conducted in accordance with the local legislations and institutional requirements. Before data collection, each potential participant was given a verbal and/or written explanation of the purpose and procedures of the study and was informed that they had the right to refuse or withdraw from the study at any time without adverse. All informants signed free and informed including permission (or not) to record for in-depth interview. Written informed consent was obtained from the individual(s) for the publication of any potentially identifiable images or data included in this article.

## Author contributions

J-PD, CBH, AV, GH, CG, and TD conceptualized the study. ÉA, MG, AV, and GH ensured data collection. ÉA, MG, AV, and GH wrote the manuscript with the support of TD, CG, CBH, and J-PD. All authors contributed to the article and approved the submitted version.
