# Supplementary material for: Effects of COVID-19 pandemic on provision and use of maternal health services in Allada, southern Benin: a local health system perspective
Source: Front Public Health. 2023 Nov 16;11:1241983. doi: 10.3389/fpubh.2023.1241983 (PMC10687162; doi:10.3389/fpubh.2023.1241983)
Supplement: Supplementary file 1 [file Table_1.DOCX]

Supplementary Material

Effects of COVID-19 pandemic on provision and use of maternal health services in Allada, Southern Benin: a local health system perspective

**Éric Akpi*, Armelle Vigan, Christelle Boyi, Marlène Gandaho, Gisèle Houngbo, Charlotte Gryseels, Jean-Paul Dossou^1^ & Thérèse Delvaux**

*** Correspondence:**  Éric Akpi, [eakpi@cerrhud.org](mailto:eakpi@cerrhud.org)

**Figure 1: Evolution of deliveries at the Communal Health Center and at the District Hospital, before, during and after the COVID-19 response in Allada, Benin**

*No deliveries at the District Hospital from March 2020 onwards*

**
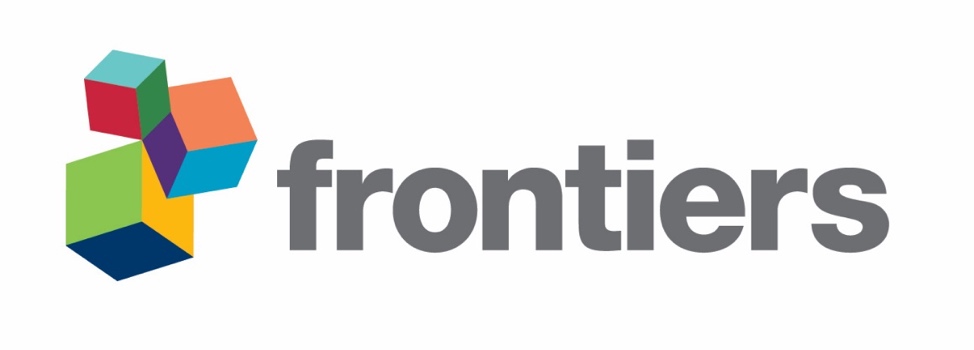
**
