# Supplementary material for: Effects of COVID-19 pandemic on provision and use of maternal health services in Allada, southern Benin: a local health system perspective
Source: Front Public Health. 2023 Nov 16;11:1241983. doi: 10.3389/fpubh.2023.1241983 (PMC10687162; doi:10.3389/fpubh.2023.1241983)
Supplement: Supplementary file 2 [file Table_2.DOCX]

Supplementary Material

Effects of COVID-19 pandemic on provision and use of maternal health services in Allada, Southern Benin: a local health system perspective

**Éric Akpi*, Armelle Vigan, Christelle Boyi, Marlène Gandaho, Gisèle Houngbo, Charlotte Gryseels, Jean-Paul Dossou^1^ & Thérèse Delvaux**

*** Correspondence:**  Éric Akpi, [eakpi@cerrhud.org](mailto:eakpi@cerrhud.org)

# Table 1: Changes induced by the COVID-19 pandemic in maternal health services provision in Allada health district, Southern Benin, 2018 to 2022

|  | **Before COVID-19**  **(Year of reference 2018)** | **During COVID-19**  **(Year of reference 2020 & 2021)** | **AFTER COVID -19 FOLLOW-UP**  **(Year of reference 2022)** |
| --- | --- | --- | --- |
| **Antenatal consultations** | **District Hospital:** Available every working day of the week from 8 a.m. to 5 p.m. | **COVID-19 treatment center:** No antenatal consultations | **District Hospital:** Available every working day of the week from 8 a.m. to 5 p.m. |
|  | **Communal Health Center:** Available every working day of the week from 8 a.m. to 5 p.m. | **Temporary district hospital (former communal health center):**  low availability of antenatal consultation (often referred to peripheral centers). | **Communal Health Center:** Available every working day of the week from 8 a.m. to 5 p.m. |
|  | **12 public peripheral health centers, 2 private health centers, 1 faith-based health center:**  Available every working day of the week from 8 a.m. to 5 p.m. | **11 public peripheral health centers, 2 private health centers, 1 faith-based health center:**  Available every working day of the week from 8 a.m. to 5 p.m. but much more in the periphery health centers. | **12 public peripheral health centers, 2 private health centers, 1 faith-based health center:**  Available every working day of the week from 8 a.m. to 5 p.m. |
| **Essential Maternal Obstetric and Neonatal Care:**   - Parenteral antibiotics - Uterotonic drugs - Parenteral anti-convulsant - Manual removal of the placenta - Remove retained products of the conception. - Assisted vaginal delivery. - Basic neonatal resuscitation - **Caesarean section delivery** - **Blood transfusion** | **District Hospital:** All functions of essential maternal, obstetric, and neonatal care (CEmONC) were available 24/7. | **COVID-19 treatment center:**  Delivery available for parturient diagnosed with severe COVID-19. | **District Hospital:** All functions of essential maternal, obstetric, and neonatal care (CEmONC) were available 24/7. |
|  | **Communal Health Center:** All functions of essential maternal, obstetric, and neonatal care (BEmONC) were available. | **Temporary district hospital (former communal health center):** Available 24/7  All functions of essential maternal, obstetric, and neonatal care (CEmONC) were available 24/7 with disturbances due to lack of space. | **Communal Health Center:** All functions of essential maternal, obstetric, and neonatal care (BEmONC) were available. |
|  | **12 public peripheral health centers:**  All functions of essential maternal, obstetric, and neonatal care (BEmONC) were available. | **11 public peripheral health centers,** 2 **private health centers, 1 faith-based health centers**  Available every working day of the week from 8 a.m. to 5 p.m. but much more in the periphery health centers. | **12 public peripheral health centers:**  All functions of essential maternal, obstetric, and neonatal care (BEmONC) were available. |
|  | **2 private health centers:**  All functions of essential maternal, obstetric, and neonatal care (CEmONC) were available 24/7. | **2 private health centers:**  All functions of essential maternal, obstetric, and neonatal care (CEmONC) were available 24/7. | **2 private health centers:**  All functions of essential maternal, obstetric, and neonatal care (CEmONC) were available 24/7. |
|  | **1 faith-based health centers**  All functions of essential maternal, obstetric, and neonatal care (BEmONC) were available. | **1 faith-based health centers**  All functions of essential maternal, obstetric, and neonatal care (BEmONC) were available. | **1 faith-based health centers**  All functions of essential maternal, obstetric, and neonatal care (BEmONC) were available. |
| **Postnatal consultations** | **District Hospital:** Available every working day of the week from 8 a.m. to 5 p.m. | **COVID-19 treatment center:** No post-natal consultation | **District Hospital:** Available every working day of the week from 8 a.m. to 5 p.m. |
|  | **Communal Health Center:** Available every working day of the week from 8 a.m. to 5 p.m. | **Temporary district hospital (former communal health center):** Available 24/7 | **Communal Health Center:** Available every working day of the week from 8 a.m. to 5 p.m |
|  | NA | **11 public peripheral health centers,** 2 **private health centers, 2 faith-based health centers**  Available every working day of the week from 8 a.m. to 5 p.m | NA |
| **Family planning** | **District Hospital:** Available every working day of the week from 8 a.m. to 5 p.m. | **COVID-19 treatment center:** No family planning | **District Hospital:** Available every working day of the week from 8 a.m. to 5 p.m. |
|  | **Communal Health Center:** Available every working day of the week from 8 a.m. to 5 p.m. | **Temporary district hospital (former communal health center):** Available from 8 a.m. to 5 p.m. with disruptions in IUD insertion or removal due to lack of materials. Referral of women IUD users to private clinics. | **Communal Health Center:** Available every working day of the week |
|  | **12 public peripheral health centers:**  Available 24/7 except IUD due to lack of equipment and lack of skills of providers. | **11 public peripheral health centers:**  Available 24/7 except IUD due to lack of equipment and lack of skills of providers. | **12 public peripheral health centers:**  Available 24/7 except IUD due to lack of equipment and lack of skills of providers. |
|  | **2 private health centers:**  Available every working day of the week | **2 private health centers:**  Available every working day of the week | **2 private health centers:**  Available every working day of the week from |
|  | **1 faith-based health center:**  No FP services available | **1 faith-based health center:**  No FP services available | **1 faith-based health center:**  No FP services available |

**
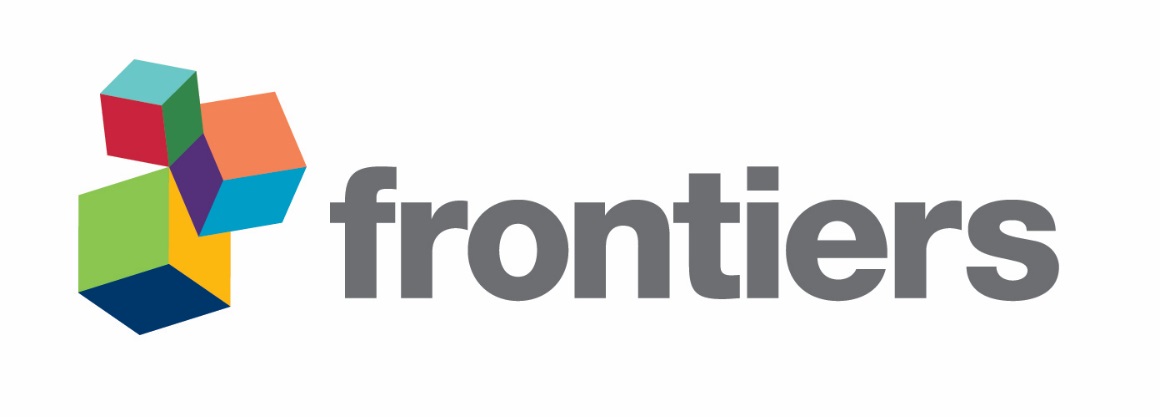
**
